# Urgently seeking efficiency and sustainability of clinical trials in global health

**DOI:** 10.1016/S2214-109X(20)30539-8

**Published:** 2021-05

**Authors:** Jay J H Park, Rebecca F Grais, Monica Taljaard, Etheldreda Nakimuli-Mpungu, Fyezah Jehan, Jean B Nachega, Nathan Ford, Denis Xavier, Andre P Kengne, Per Ashorn, Maria Eugenia Socias, Zulfiqar A Bhutta, Edward J Mills

**Affiliations:** Department of Experimental Medicine, University of British Columbia, Vancouver, BC, Canada; Epicentre, Paris, France; Clinical Epidemiology Program, Ottawa Hospital Research Institute and School of Epidemiology and Public Health, University of Ottawa, Ottawa, ON, Canada; Department of Psychiatry, Makerere University, Kampala, Uganda; Department of Paediatrics and Child Health, Aga Khan University, Karachi, Pakistan; Department of Medicine and Center for Infectious Diseases, Stellenbosch University, Cape Town, South Africa; Department of Epidemiology and Department of International Health, Johns Hopkins Bloomberg School of Public Health, Baltimore, MD, USA; Department of Epidemiology and Department of Infectious Diseases and Microbiology, University of Pittsburgh Graduate School of Public Health, Pittsburgh, PA, USA; Centre for Infectious Disease Epidemiology and Research, School of Public Health and Family Medicine, University of Cape Town, Cape Town, South Africa; Department of Pharmacology and Division of Clinical Research, St John’s Medical College, Bangalore, India; Non-Communicable Diseases Research Unit, South African Medical Research Council, Cape Town, South Africa; Faculty of Medicine and Health Technology, Tampere University, Tampere, Finland; Fundación Huésped, Buenos Aires, Argentina; British Columbia Centre for Substance Use, Vancouver, BC, Canada; Department of Medicine, University of British Columbia, Vancouver, BC, Canada; Centre for Global Child Health, Hospital for Sick Children, Toronto, ON, Canada; Institute of Global Health and Development, and Center of Excellence in Women and Child Health, Aga Khan University, Karachi, Pakistan; School of Public Health, University of Rwanda, Kigali, Rwanda; Department of Health Research Methods, Evidence, and Impact, McMaster University, Hamilton, ON, Canada; Cytel, Vancouver, BC, Canada

## Abstract

This paper shows the scale of global health research and the context in which we frame the subsequent papers in the Series. In this Series paper, we provide a historical perspective on clinical trial research by revisiting the 1948 streptomycin trial for pulmonary tuberculosis, which was the first documented randomised clinical trial in the English language, and we discuss its close connection with global health. We describe the current state of clinical trial research globally by providing an overview of clinical trials that have been registered in the WHO International Clinical Trial Registry since 2010. We discuss challenges with current trial planning and designs that are often used in clinical trial research undertaken in low-income and middle-income countries, as an overview of the global health trials landscape. Finally, we discuss the importance of collaborative work in global health research towards generating sustainable and culturally appropriate research environments.

## Introduction

The field of global health prioritises improving health and achieving health equity for all. Research in this area is focused on improving health outcomes and addressing inequities in low-income and middle-income countries (LMICs).^1,2^ LMICs continue to have comparatively higher rates of and burden from infectious diseases and other communicable diseases (eg, HIV, malaria, diarrhoeal diseases, and sepsis) than do high-income countries (HICs). Among many health challenges, shorter life expectancy and higher rates of maternal and child mortality in LMICs, relative to HICs, have delayed their developmental and economic potential.^3–5^ Thus, interventions to reduce disease and improve health in LMICs, particularly, should be a global priority.

Progress has been made in the prevention and treatment of infectious diseases and other communicable diseases, with randomised clinical trials (RCTs) having been used to generate evidence on the effectiveness of important therapeutics. RCTs are considered robust methods for evaluating the effectiveness of therapies because these studies fulfil the primary assumption of statistical testing—ie, the equality of treatment by minimising selection bias and creating groups that are comparable in prognostic factors—thereby establishing a causal effect of the treatment on the outcome.^6,7^ RCTs are an important methodological tool in global health research for generating high-level evidence to inform the development of context-specific and international guidelines on preferred interventions that can be delivered at scale to populations in need.^5^

Although the challenges in reducing the burden of communicable diseases in LMICs are considerable, there have been important demographic and epidemiological shifts occurring rapidly in LMICs. The global population is estimated to increase from approximately 7·7 billion people in 2019, to 8·5 billion by 2030, and further to 9·7 billion people in 2050.^6^ The most rapid growth is projected to occur in sub-Saharan Africa, with the population size expected to double by 2050.^6^ With the increasing life expectancy in LMICs, there will be a consequent increase in the burden of non-communicable diseases (NCDs), because these diseases are more common in adults older than 65 years.^7–9^ This rapid change in population size and demographics, in addition to the rising burden of NCDs, presents many challenges for meeting the Sustainable Development Goals.^8–10^ A change in approach is needed to rapidly answer research questions that can accompany the changes in demographics and disease burden in LMICs, where resources are limited and health research capacity is low. In the current era, efficient research designs that can answer multiple complex research questions simultaneously with a minimised sample size and trial duration will be beneficial, but there are also many logistical and methodological barriers to overcome before such an approach is a reality.

This paper in the Series reviews the close connection of global health with methodological research. We summarise the current landscape of global health trial research, the limits of conventional approaches to trials, and challenges in trial planning.

## History of RCTs

Global health research is inherently connected to clinical trials. Sir Austin Bradford Hill advocated for RCTs for at least a decade before the first peer-reviewed RCT in the English language was published in 1948.^11–13^ According to Bradford Hill, the concept of randomisation was extraneous to the individuals of the medical community in the UK, many of whom rejected the notion at the time.^13^

The 1948 streptomycin trial for pulmonary tuberculosis from the UK Medical Research Council^11^ changed the landscape of medicine and public health. The trial was done shortly after World War 2, a time of immense resource limitations and widespread illness and injuries.^13^ The war had effectively stripped the UK of financial resources and the government could only afford to purchase streptomycin, a new drug for treating tuberculosis, for only 55 patients.^13,14^ Economic hardship was a dominant reason that made this RCT possible. The high burden of tuberculosis and scarce drug resources justified the UK Medical Research Council in using an RCT, to ensure the fair distribution of a scarce drug supply and to obtain a reliable answer regarding the effectiveness of streptomycin for treating tuberculosis.^11,13,15^

In the seven decades since, the pharmaceutical industry, particularly in the area of oncology research, has made several important advancements in statistical analysis, simulation methods, and outcome assessments for innovative designs, such as adaptive trial designs and master protocols.^16–19^ These innovative designs have also been used, although scarcely, in specialised fields, such as clinical neonatology.^19,20^ However, uptake of these methods has been poor in global health research,^21,22^ much like the situation in which the medical community was initially reluctant to embrace the concept of randomisation less than a century ago. With the increasing challenge of the double burden of NCDs and communicable diseases in the context of global health, questions to be answered are manifold and more complex; however, human resources and research infrastructure in LMICs are limited. Adopting methodological innovations to clinical trial design and implementation has the potential to bring the efficiency needed to global health research.

## Current landscape of trial research in global health

Although any definition of global health is contentious, in this Series, we will refer to global health research as any clinical research undertaken in a collaboration between researchers in HICs and LMICs, in which most intended monetary funding comes from outside of the country that the research is conducted in. This definition recognises the potential power imbalance among researchers and among sponsoring institutions. Throughout this Series, we discuss several limitations of current, possibly inefficient and often outdated, approaches to trial design, but it is important to note that these criticisms apply equally to all those working in global health research, including (or potentially predominantly) researchers in HICs designing these studies and the funders sponsoring them, in light of these power imbalances.

### Mismatch between research efforts and disease burden

**Figure 1:**
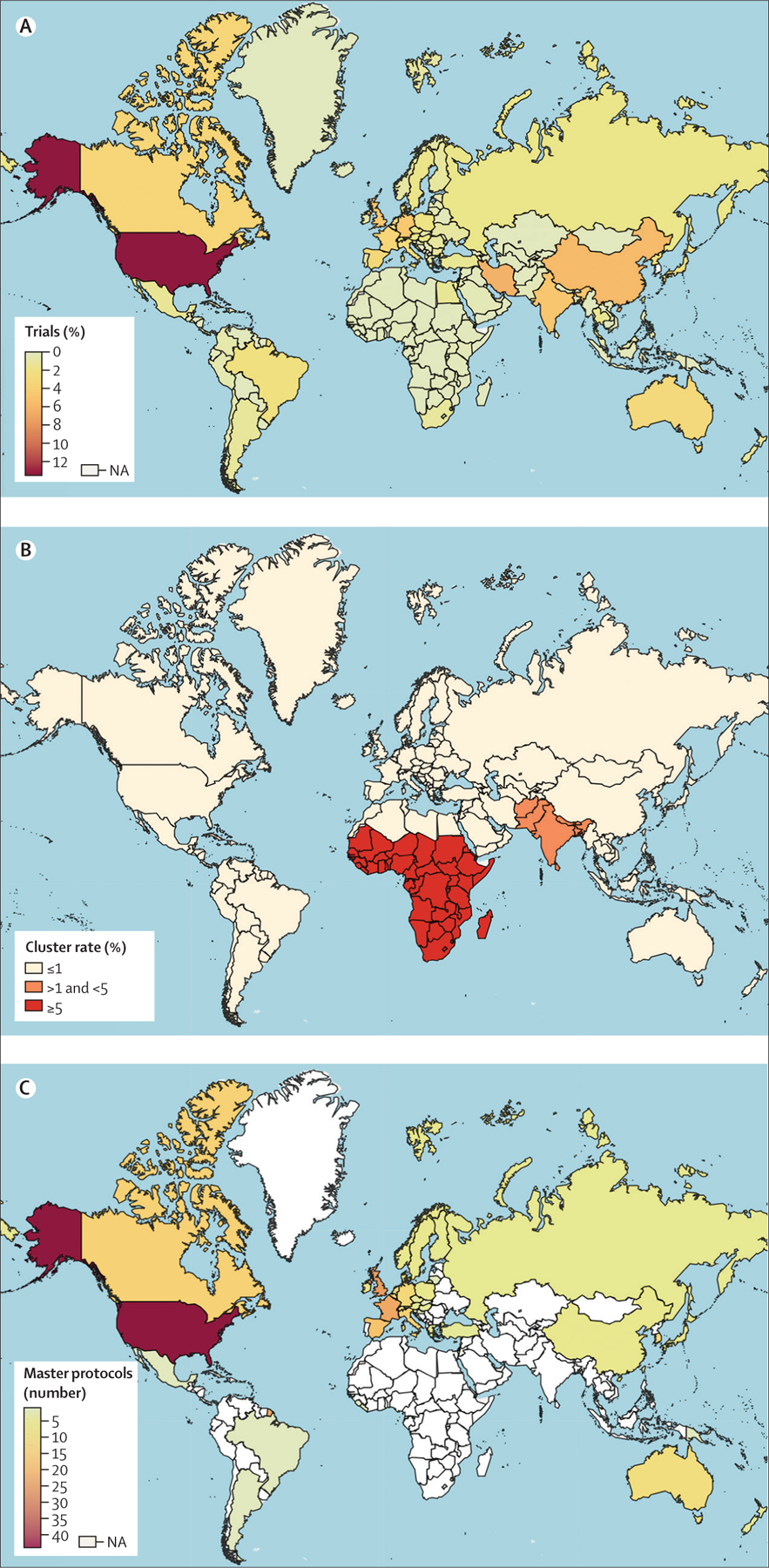
Global overview of clinical trial research (A) Percentage of registered randomised clinical trials worldwide. (B) Percentage of trials that are cluster-randomised, by country, from the WHO International Clinical Trials Registry Platform from Jan 1, 2010, to Nov 5, 2019. (C) Number of master protocols registered or undertaken worldwide as of Dec 11, 2019.^21^

There is a well recognised mismatch between research efforts (eg, number of clinical trials) and disease burden.^23^ Despite public health efforts to minimise disparities in health, research efforts have largely been focused towards the interests of HICs (eg, the USA and the UK), particularly in markets for which high commercial interests and sustained public funding exist. Several studies have shown that most clinical trial research is undertaken in HICs.^23–26^ According to the WHO International Clinical Trial Registry, as of Nov 5, 2019, RCTs in LMIC settings have only accounted for 109 713 (32%) of 342 854 RCTs registered worldwide (figure 1). Despite 24% (1·8 billion people estimated for 2019) of the global population currently residing in south Asia, this region is the setting of only 5% (n=17 777) of all RCTs worldwide.^6^ Sub-Saharan African countries (which had a population of 1·1 billion people in 2019) are the setting of an even smaller proportion of RCTs, at 2% (n=5756).^6^

The low number of RCTs represents a missed opportunity for LMICs and suggests the need for more clinical trial research in global health. When global health clinical trials are undertaken, they are often done with insufficient funding to support sustained development of research infrastructure and human resources in the LMICs in which they are set. To maintain clinical trial research efforts in global health over the long term, the ability to sustain research infrastructure and human resources for local settings through consolidated and minimised single-use research activities related to trial planning and execution is crucial.

Efforts to support local ownership and coproduction of health research in LMICs are rare.^27^ In key topical fields of global health research, such as HIV and AIDS, malaria, and tuberculosis, there is a low first-authorship of researchers from LMICs on publications, raising a concern of rare opportunities for research, career advancement, and capacity building for LMIC researchers.^28^

### Importance of location and context

The world is often dichotomised into the industrialised world versus the developing world. In global health research, there are often tendencies to ignore the different contextual factors between different LMICs and to search for common interventions that will improve health across multiple countries in many diverse populations.^29^ However, the reality is that there is often no one-size-fits-all solution because there is substantial heterogeneity between and within different countries.^30^

Different biological and socioeconomic factors of the trial location should be considered during the planning stage, to determine whether the research question is being asked in the right setting. For instance, despite a total of 168 460 neonates having been studied across 12 trials in LMICs, a 2017 Cochrane review by Haider and colleagues^31^ reported inconclusive evidence for neonatal vitamin A supplementation on infant mortality. There were conflicting findings between different individual studies that had importantly different rates of maternal vitamin A deficiency and infant mortality.^31^ It is ill-advised to undertake clinical trials on vitamin A supplements among populations with low or no vitamin A deficiency. If these resources had instead been allocated to undertaking trials in regions where the prevalence of vitamin A deficiency is high, it is likely that there would be more conclusive evidence to support or refute the benefits of vitamin A supplementation on infant mortality.

It is beneficial if biological samples are collected, to better understand the biological mechanisms of different interventions. Biological information can become especially important when the statistical assessment of the clinical outcomes show no difference. Collection of biological samples allow for mechanistic analyses to be undertaken alongside the main analyses of clinical trials, to better understand what happens biologically when an intervention is given. If laboratory analyses can show no biological associations, negative trial results could mean that the intervention probably does not have the initial hypothesised effects.^29^

In addition to biological analyses, consideration of clinical events that occur after a given intervention is initiated (ie, intercurrent events) is important. Intercurrent events, such as discontinuation of intervention, switching intervention, and use of rescue medications, are often handled with simple intention-to-treat (ITT) analyses.^32^ The ITT principle is intended to measure the effectiveness of intervention in real-world conditions, in which participants will not adhere perfectly to the protocol.^33,34^ However, the intercurrent events can increase the variability of the data and make interpreting the study findings difficult. For instance, with the ITT principle, the discontinuation of an intervention is often ignored in the main analysis even though this action will affect the clinical outcomes of the participants in the study. Given that ITT analysis is used as the main primary analysis, different analytical strategies to handle discontinuation of intervention or other intercurrent events as sensitivity analyses are beneficial to explore the robustness of the ITT analysis.^33,35^

### Affinity towards conventional designs

In global health research, conventional fixed trial designs are most often used for clinical research. Fixed trial designs refer to a type of design in which the trial data are analysed only once, when the trials are finished, after determining a sample size a priori.^36,37^ Fixed trial designs do not plan for any modifications to major design components (eg, sample size, allocation ratio, and number of interventions) throughout the trial.^36–38^ In clinical trials, data are accumulated over time, and some clinical trials might take years to complete. Fixed trial designs do not permit learning during the trial from the accumulating trial interim data because the interim data are not analysed throughout the trial. Investigators usually make assumptions about the population, interventions, outcomes, and other trial parameters on the basis of information that is available at the planning stage, and continue these assumptions throughout the trial until the last participant has completed their follow-up.

It is difficult to determine why methodological advancements in clinical trials have been slow to develop or gain acceptance in global health research. Advocacy for clinical trial education in LMICs has predominantly occurred via academic medical journals. The leading journals have published widely on methodological issues advocated by expert groups, such as the Consolidated Standards of Reporting Trials, Grading of Recommendations, Assessment, Development, and Evaluation, and Cochrane collaborations, which have historically placed a primacy on individual quality indicators of clinical trials. These quality indicators have examined issues that can affect the precision of trial estimates and their likelihood of introducing bias. However, scoring the quality of clinical trials can be misleading and potentially hazardous if clinical decisions or public health decisions are influenced by evidence that could later be contradicted.^39^ Many of the methodological features that are considered key to determining the quality of a trial, including blinding, reporting of ITT analysis, allocation concealment, and sequence generation, have clear benefits, but there are issues with these features when evaluating whether randomised trials with a different design should be considered in policy making; a more nuanced approach is needed, given that there have been inconsistent findings as to whether these features are actually important to biasing study results.^40,41^ However, these features are still routinely taught and are embedded in WHO guideline evaluations to determine whether guidelines provide strong evidence or not. The global health community could benefit from gaining an understanding of more innovative methods that are now available.

**Figure 2:**
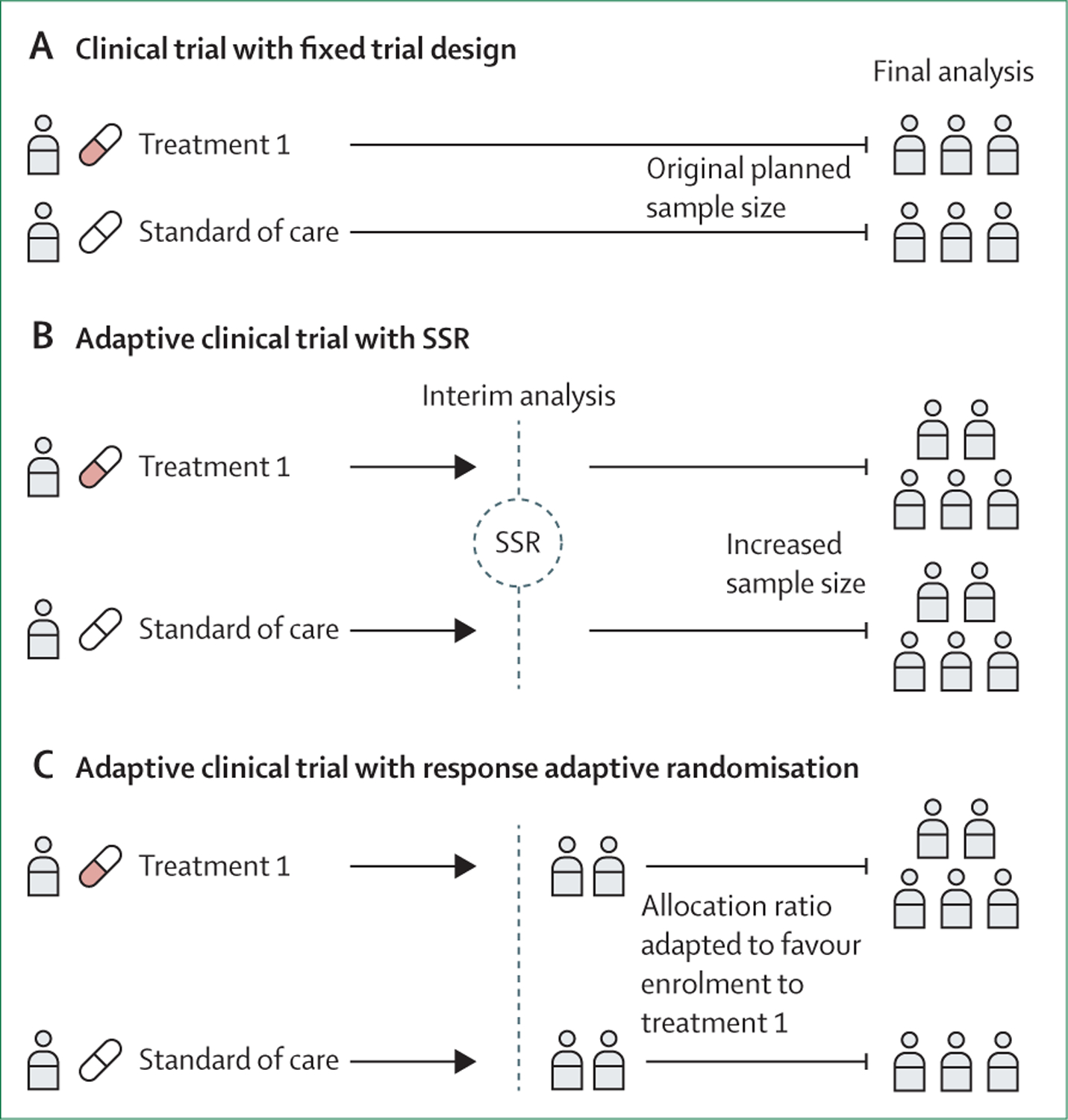
Conventional fixed trial designs and common adaptive trial designs (A) A two-arm randomised clinical trial with conventional fixed trial design. (B) A two-arm trial with SSR. If the first interim analysis shows worse results than expected, an SSR can be carried out by use of the interim results. An SSR is not permitted in a traditional non-adaptive trial, so even when the original planned sample size is reached, the trial might be underpowered. If SSR is permitted, the sample size could be increased to ensure that the trial is adequately powered. (C) A two-arm trial with response adaptive randomisation. The response adaptive randomisation design allows for preferential assignment of interventions that show favourable interim results. In this example, the response adaptive randomisation design allows for an increased allocation ratio to treatment 1 based on the interim results. SSR=sample size reassessment.

In the past few decades, important methodological advancements, particularly in adaptive trial designs, have sought to offset limitations that conventional trial designs can pose.^17,42^ An adaptive trial design, an extension of conventional fixed trial designs, is a type of trial design that allows for prespecified modifications (or adaptations) to the trial design during the trial, including plans for interim evaluations and decision rules.^16,17,37^ Adaptive trial designs do not necessarily impose modifications to a trial: if prespecified decision rules are not met according to the trial data, the trial would continue without any adaptations and function as if the trial had a fixed trial design (figure 2). In brief, an adaptive trial design is a data-driven approach to a clinical trial investigation that allows trial modifications before an a priori sample size target has been reached.

Common examples of adaptive trial designs include group sequential designs and sample size reassessment.^17,37,38^ A group sequential design is a type of design that allows for early stopping with stopping rules, usually based on a frequentist statistical metric in test statistics (typically p value boundaries).^17,43^ If the interim data assessment shows crossing of stopping boundaries, then the trial might stop under a group sequential design. With more frequent observations, inflation of type I error rates can occur (multiplicity), especially without statistical adjustments. In a commonly used group sequential design, such as the O’Brien-Fleming approach, the stopping boundaries are set more stringently with more interim analyses to preserve a significance level for the final analysis that is close to the significance level of a single analysis (eg, 5% α).^43^ Sample size reassessment is another type of adaptive trial design that allows for an increase in sample size based on interim data.^17,38^ Sample size reassessment was developed to mitigate risks for false-negative findings.

Although adaptive trial designs have received substantial attention in recent years, they are not without limitations. Every clinical question deserves a thorough investigation and consideration of the pros and cons when selecting design options and there should be no default design choice. An efficient trial design for one research question can be inefficient for another. Undertaking smarter trials requires a thorough consideration of multiple candidate designs during the planning stage, ideally with the use of statistical simulations to weigh the efficiencies of different designs. Further discussion of adaptive trial designs and case studies are provided in the second^44^ and fourth^45^ papers of this Series.

### Inadequate planning practices

Often in global health research, very little information is available regarding epidemiology and infrastructure in the context of the investigation, which can make trial planning difficult. Sample size calculations are a key challenge due to the unfamiliarity with regard to local epidemiology and expected treatment effects or accepted minimally clinical important difference. The recommended process of determining a realistic or important target difference between treatments has previously been described in the difference elicitation in trials (also known as DELTA^2^) guidance.^46^

Sample size calculations are a simplistic form of simulating a trial, and sample size calculations for RCTs with dichotomous outcomes require prespecification of operating characteristics, event rates in the control and intervention groups (ie, desired or expected effect sizes), and the rate of loss to follow-up. In most research areas within global health, operating characteristics are usually selected by convention at 80% statistical power and 5% type I error rate. There are no conventions for selecting event and treatment effect parameters when investigating dichotomous or other outcomes (eg, continuous and count outcomes). Clinical trials are often underpowered even when the planned sample size is reached due to erroneous assumptions used for sample size calculations, such as lower incidence of events than planned and lower treatment effects than anticipated. At the other extreme, the trial could be overpowered and too many participants might be recruited into a given trial, wasting time and resources.^38^ Consequently, the development of essential guidance might be delayed because another trial is required, or because the original trial has taken longer than necessary.

Appropriate sample size calculations are dependent on correct assumptions of design parameters. Often in global health research, clinical trials are planned on the basis of nationally representative cross-sectional survey data, such as Demographic and Health Surveys and Multiple Indicator Cluster Surveys, which monitor and evaluate programme implementation at a national level.^47^ Although these cross-sectional surveys are valuable for these purposes, their use for trial planning, including sample size calculations, can be limited. These surveys fail to show temporal variability that might exist in disease severity because these cross-sectional surveys are not undertaken annually; Demographic and Health Surveys are typically carried out every 5 years^48^ and Multiple Indicator Cluster Surveys are carried out every 3 years.^49^ Additionally, these surveys report on the national or regional prevalence of disease, and not the incidence, and can miss important geographical or demographic heterogeneity.

Assumptions used for sample size calculations can also be based on findings from previous studies. The use of a thorough literature review is important to identify reliable estimates; however, making such assumptions by extrapolation from previous studies can also have limitations. Researchers must account for how similar (or dissimilar) a previous study is to the trial being planned, including the recency of the study findings, or geographical and demographic differences. Even when there are recently undertaken studies with geographical and demographic similarities, there are still possibilities of having unexpected findings (panel).

However, sample size calculations are only a small part of efficient trial planning. A thorough consideration of all accessible scientific knowledge and exploration of multiple scenarios to anticipate potential risks and a range of expected results is also required in the planning stage.^37^ This approach can be achieved by use of computational simulations of study results under various scenarios that test the fragility of assumptions and weigh the pros and cons of different candidate design strategies for smarter trial planning.^53^ Despite its importance, only a few examples of clinical trials incorporating simulations exist in global health research.^54–56^

### Cluster trial designs

Cluster-randomised trials are a type of clinical trial that involve randomisation of groups or clusters of participants, rather than individuals.^57^ Cluster trials are appropriate for settings in which the use of individual randomisation is not feasible due to the nature of interventions being evaluated or in which substantial contamination is unavoidable. Cluster trials might also be used in cases for which the final aim is to evaluate the effectiveness of interventions at a mean cluster level.^57^ Therefore, such trials are useful for evaluating the effectiveness of interventions or practices at the population level. For instance, in the early 1990s, several cluster trials were undertaken to determine the effects of insecticide-treated bednets for malaria prevention,^58–62^ because testing these bednets in patient-level RCTs would not have been feasible. These cluster trials have provided strong evidence for the effectiveness of insecticide-treated bednets, a core component of malaria prevention efforts in LMICs.^63^

Compared with HICs, cluster trials are more frequently undertaken in LMICs even though there are far fewer RCTs undertaken (figure 1). In HICs, cluster RCTs are undertaken relatively infrequently, with only 1657 (0·72%) cluster trials of all 231 477 trials documented; by contrast, the proportion of cluster trials is more than doubled in south Asia (308 [1·73%] cluster trials of all 17 777 trials) and eight times higher in sub-Saharan Africa (354 [6·04%] cluster trials of all 5859 trials). Although there are instances in which cluster trials are appropriate (eg, when interventions cannot be delivered at the individual level), it is important to note that cluster trials can be expensive to carry out due to their statistical inefficiencies. Because the observations of individuals within the same cluster are usually correlated, there are statistical inefficiencies associated with cluster randomisation. To achieve statistical control of the probability of false-negative risks and false-positive risks (eg, 80% statistical power and 5% type I error rate), cluster trials require a much larger number of participants than individually randomised trials. Detailed discussions of cluster trial designs in the context of global health are provided in the third paper^64^ of this Series.

### Factorial trial designs

Factorial clinical trials are a type of clinical trial that simultaneously test the effect of two or more interventions with the use of various combinations of interventions.^65^ For instance, in a two-by-two factorial trial design, participants or clusters are randomised to one of the four combinations of two interventions (eg, A and B); the combination of these intervention strategies (A alone, B alone, and A plus B combined) can be compared against a control arm that does not receive intervention A or B. Factorial trial designs can be appealing because two or more interventions can be assessed at the same time in the same population simultaneously.^66^ Assuming that there is no interaction between the interventions, factorial designs can be an efficient way to test multiple interventions. Factorial designs can also allow for the testing of treatment interactions, but, depending on treatment interaction effects, these designs can result in sample sizes often four times higher than a comparable two-arm trial.^66^ To undertake efficient factorial clinical trials, it is important to have a reliable estimate of the treatment interaction effects. Such estimations can be challenging because reliable estimates regarding treatment interaction often do not exist. Treatment interaction estimates are essential for determining sample size requirements because interactions can substantially increase the needed sample size. Implementing factorial trials can also be more operationally challenging than two-arm trials because such trials usually involve multiple arms.

## Building on long-term research infrastructure and capacity in global health

There is a need to build long-term research infrastructure and capacity in LMICs for long-term sustainability.^67,68^ In the context of global health, limited infrastructure and capacity in some regions can pose a challenge in carrying out clinical research.^69^ However, it is important to recognise that some regions have not been given a long-term opportunity to build and sustain an infrastructure that would also allow for local training and professional development to be improved. There is a need to grow a large network of competent research groups by dedicating long-term funding for infrastructure and professional development, while ensuring that LMIC investigator-initiated or institution-initiated trials that aim to provide answers to local priorities have precedence.

The adoption and application of data-driven methods (eg, adaptive trial designs) has been scarce in global health research,^70–74^ despite their potential to save resources, improve the chance of identifying effective interventions, and address certain ethical concerns. Additionally, long-term trials that test multiple interventions with the use of a common control under a master protocol framework—perhaps the most efficient of all trial designs—have mainly been done in HICs, and not in LMICs (figure 1).^21,22^ A master protocol refers to a framework in which clinical trial research is undertaken with an overarching design that has been developed to evaluate multiple hypotheses with the aims of improving efficiency and establishing uniformity through standardisation of procedures in the development and evaluation of interventions.^18,75,76^ Master protocols can differentiate into multiple parallel substudies to include standardised trial operational structures, patient recruitment and selection, data collection, analysis, and management.^18,75,76^ In LMICs, adopting the framework of master protocols could be one way forward towards improving the coordination and sustainability of clinical research efforts in the global health field.

Clinical trial research in global health can often be fragmented and uncoordinated with a preponderance of short-term two-arm trials that have low statistical power and are small in scale. This preponderance of two-arm trials that have low statistical power can be attributed to a scarce funding availability, in which investigators compete for a small pool of funds. Funders rarely collaborate, and there might be little incentive for greater collaborations of researchers in geographical regions beyond their existing professional networks. This norm of short-term funding gives little room to consider building local infrastructure and human expertise. When these short-term trials finish, the infrastructure often disappears along with the data generated from research and the investigators who brought the funds from HICs, and investigators based in the country and who actively participated in the research are frequently omitted in peer-reviewed publications.^28,77–79^

To build a lasting research capacity in LMICs in a sustainable manner, equitable collaboration with the local researchers is needed. This collaboration, of course, should include fair equitable representation of local researchers from peer-reviewed publications and fair allocation of funds with local institutions. Collaboration between existing or newly established clinical trial networks (eg, AIDS Clinical Trials Group and European and Developing Countries Clinical Trials Partnership) should also be fostered and incentivised to allow for the exchange of ideas, supervision, and mentorship between different places, markets, or people that can facilitate the securing of resources and training, as well as educational opportunities in innovative trials designs, to ensure that efficient clinical trial research can be undertaken in a sustainable manner for global health.^80–82^

For example, maternal, newborn, and child health is one area in the field of global health that has seen fragmented and uncoordinated efforts, despite having clear and specific global targets set of reducing low birthweight by 30% and stunting in children younger than 5 years by 40% globally.^83–85^ The clinical trial evidence remains weak for interventions aimed at reducing adverse birth outcomes and child stunting in the first 1000 days of life.^86–88^

A 2019 systematic review identified 169 RCTs in LMICs (comprising 302 061 participants) that evaluated the comparative efficacy of interventions under multiple domains of micronutrient and balanced energy protein or food supplements, deworming, maternal education, water sanitation, and hygiene during pregnancy, exclusive breastfeeding, and complementary feeding periods (ie, the first 1000 days of life; appendix p 2).^89^ For this key global health priority, 86 (51%) of 169 of these trials were of 26 weeks in duration or shorter, 69 (41%) of 169 of the trials recruited fewer than 500 patients, and 101 (60%) of 169 of the trials were not statistically conclusive on important clinical outcomes.^89^

Most of these trials were either single-centre trials or the trials had recruited patients from a few centres in the same country or region, usually around the capital city or a nearby region. Because clinical trials are often undertaken to estimate the population-level effects of given interventions, the norm of carrying out single-centre trials can be problematic for global health research. Geographical variation cannot be shown when trials are undertaken in a small number of centres and random effects of interventions arising from geographical variability cannot be estimated (appendix pp 3–4). If the intervention being investigated truly has no effects at the population level (null effects), the intervention might still show positive treatment effects if the trial is undertaken at one centre by play of chance. Trials carried out in this way would result in an inflated type I error rate because, in these single-centre trials, other locations that would show negative effects (which would cancel out the positive effect from the one centre with this finding) are omitted. Conversely, if the intervention does have a true positive treatment effect at the population level, an opposite effect (ie, failing to show an effect for an effective treatment) might be observed due to the use of a location that shows null or negative effect by chance. Thus, undertaking single-centre trials might result in a lower statistical power than multicentre trials.

All of these trials used fixed trial designs, even though well planned interim evaluations could have ended the trials for reasons of superiority or futility and saved resources for other research. Interim analyses can also help protect participants. Early stopping for superiority could translate to early dissemination of effective interventions, and early stopping for futility could minimise unnecessary exposure to participants. The current evidence would arguably be stronger if the resources that were invested towards these multiple small two-arm clinical trials had been diverted into a few larger multi-arm trials with adequate statistical power and trial planning. Combined resources could also have allowed for the collection of biological samples and sociocultural data to better characterise the study context and, thus, allow for an improved understanding of the mechanism of interventions.^29^ We continue the discussions for interim analyses for early stopping and the framework of master protocols for global health in further detail in the second^44^ paper of this Series.

## Conclusion

Clinical trial research has had a strong connection to global health that aims to improve health equities for populations that have a high burden of disease. Clinical trial research has identified many essential interventions for communicable diseases in LMICs but, with many LMICs rapidly transitioning into an NCD era, and with shortages of necessary funding for research, serious challenges await. The global health trial landscape has remained largely static over the past 70 years, with few examples of new methodological innovations being adopted. In the second paper of this Series, we outline new methods and tools that can be used to improve the efficiency and quality of global health clinical trials.

## Supplementary Material

Supplementary Material

## Key messages

- Low-income and middle-income countries face the most rapidly occurring demographic and epidemiological transitions globally that threaten sustainable global development.
- Clinical trial research has an inherent connection with global health research that aims to answer important questions for populations in need, but there is a mismatch in clinical trial efforts and disease burden globally. Of the 324 854 randomised clinical trials that have been registered from 2010 to 2019, our analysis has shown that only 17 777 (5%) of the trials were set in south Asia and only 5756 (2%) of the trials were set in sub-Saharan African countries.
- Clinical trials that are undertaken in low-income and middle-income countries can be improved by promoting new methodological innovations that can be leveraged to improve the ability of the local regions across low-income and middle-income countries to sustain research infrastructure and human resources for long-term gains.

## Panel: A rural Tanzania-based cluster trial studying the efficacy of topical repellent on malaria transmission (ISRCTN92202008)

Between July, 2009, and August, 2010, a placebo-controlled cluster trial was undertaken in a rural village in Tanzania (Mbingu village of the Ulanga district) to evaluate the efficacy of topical repellent on reducing malaria transmission.^50^ After sample size calculation, it was determined that enrolling 10 clusters of 47 households (each with five family members) would yield 90% statistical power at a 5% type I error rate, assuming a 30% control event rate of evening malaria transmission (primary outcome) and a treatment effect size of 80% risk reduction.^50^

Several assumptions were made to justify the 30% control event rate for sample size calculation, given that there were no available estimates of malaria transmission in this region.^50^ The trial investigators assumed a malaria incidence rate of 1·0 malaria case per person per year on the basis of available estimates of fever incidence rate among children younger than 5 years (3·2 cases per person per year^51^), under the assumptions that only a proportion of fevers are caused by malaria and that the village population would be less prone to fevers than children.^50^ Because it has been previously estimated that 30% of mosquito bites occur in early evenings,^52^ the trial investigators justified the control event rate of 30% by combining the assumed malaria incidence rate with the proportion of evening mosquito bites.^50^

During the trial, Tanzania had a drought that reduced the malaria transmission rate, which led to a lower control event rate, meaning that the trial became underpowered due to the drought. This trial did not identify any benefits of topical repellent in malaria transmission reduction. The investigators have attributed the negative effects to the lower malaria infection rate, because only a 6% infection rate was identified in the trial instead of the anticipated 30% infection rate.^50^ Despite the unexpected challenges that occurred, this trial continued until its intended completion date as originally planned, with 4426 individuals being enrolled.^50^ When observed control event rates are considerably lower than the rates anticipated for initial trial planning, clinical trials, in turn, will be underpowered and there will be little (or no) promise of providing conclusive answers. To anticipate for such instances, prespecifying mechanisms of applying a futility analysis through adaptive trial designs could be useful.
