# Supplementary material for: Urgently seeking efficiency and sustainability of clinical trials in global health
Source: Lancet Glob Health. Author manuscript; Available in PMC 2021 Sep 8. (PMC8424133; doi:10.1016/S2214-109X(20)30539-8)
Supplement: Supplementary Material [file NIHMS1720586-supplement-Supplementary_Material.pdf]

THE LANCET
Global Health

## Supplementary appendix

This appendix formed part of the original submission and has been peer reviewed. We post it as supplied by the authors.

Supplement to: Park JJH, Grais RF, Taljaard M, et al. Urgently seeking efficiency and sustainability of clinical trials in global health. *Lancet Glob Health* 2021; **9:** e681–90.

**Supplementary for “Global health clinical trials: Urgently seeking efficiency and sustainability”**

*Supplementary Figure 1: A survey of LMIC-based trials in maternal, newborn, and child health research*

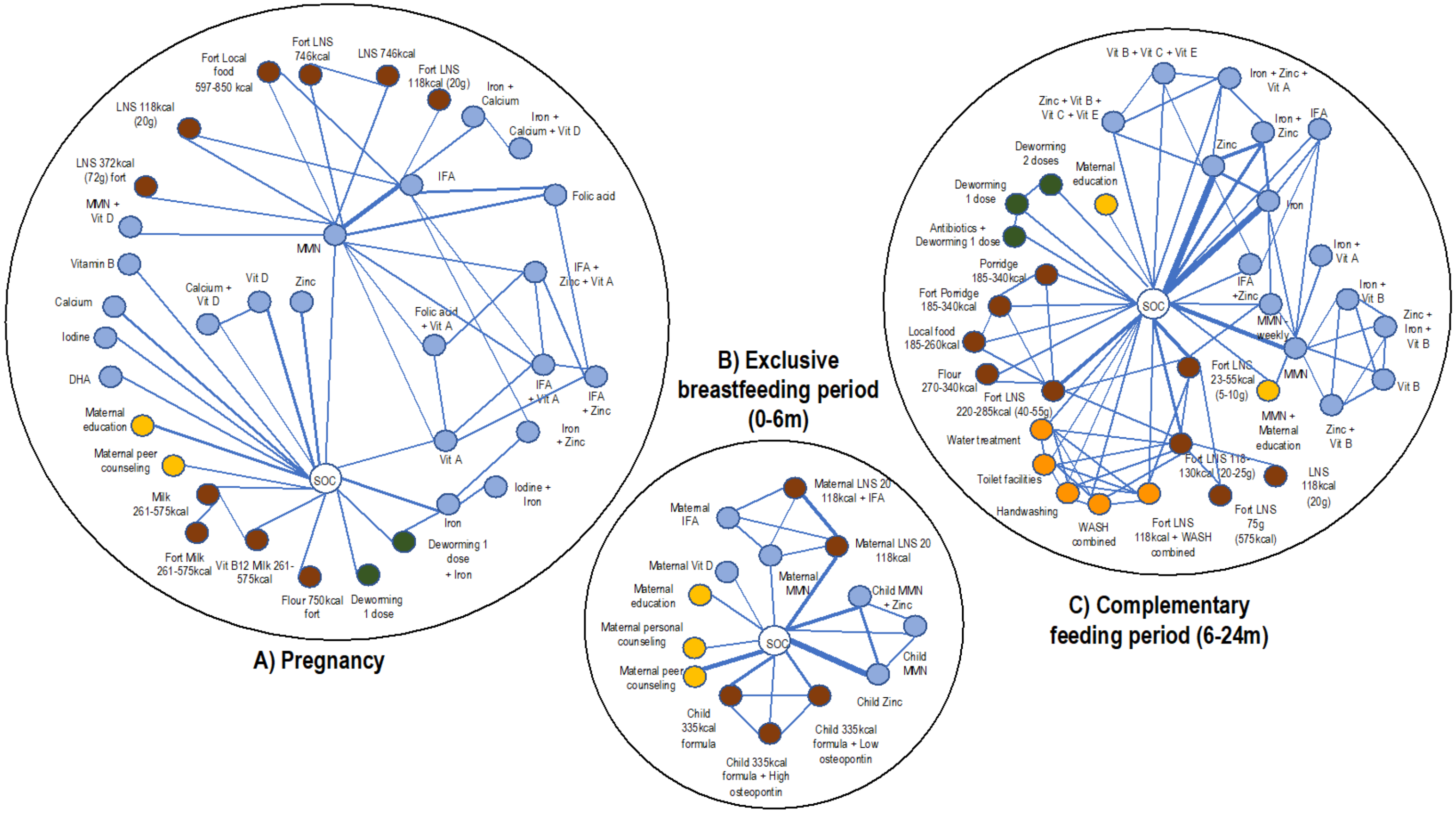


**Legend**: Each node represents an intervention with each line representing interventions that have been compared directly in at least one clinical trial. The width corresponds to the number of trials.

*Supplementary Figure 2: Comparison of single- and multi-centre clinical trials*

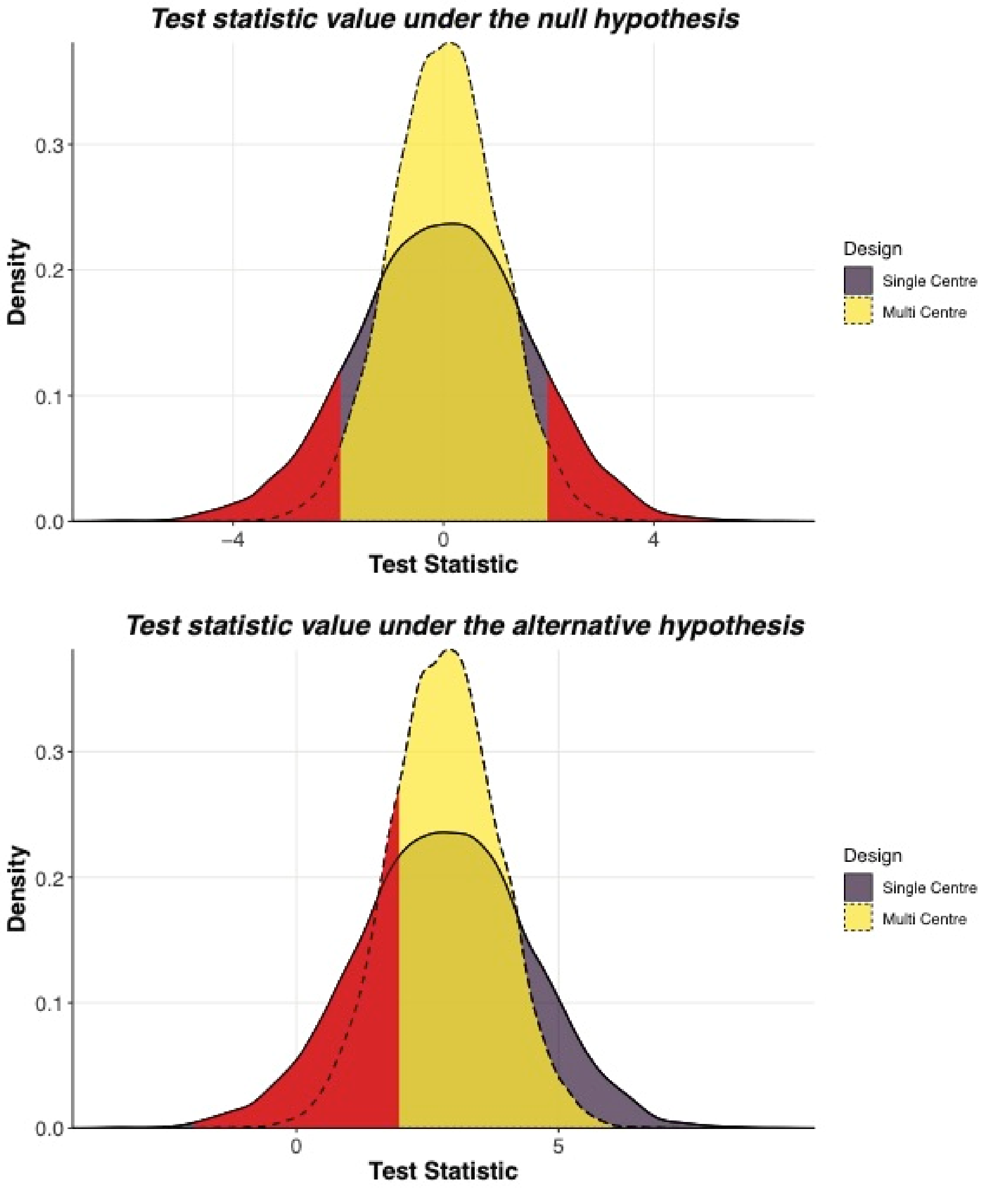


**Legend**: This figure illustrates the distribution of test statistics of clinical trials conducted in a single centre versus multi-centres under the null hypothesis (upper figure) and alternative hypothesis (bottom figure). The red area in the upper figure highlights the size of false positive (type I error) rate of the respective designs; the red area in the bottom highlights the size of false negative (type II error). Consider the following scenario: Suppose that due to a combination of the nature of the outcome and the conditions targeted by the trial, the expected response would vary should separate trials be conducted at various centres. This variability leads to a mixed effect model, with treatment variable as the fixed effect and the centre as the random effect. However, because the trial is conducted at a single centre, the random effect component cannot be estimated, and the true variability of the response is

underestimated. Consequently, if the treatment under investigation is non-efficacious (on average) but the centre chosen for the trial is one where the treatment effect is above average (i.e. has a positive random effect), the treatment will flatter to deceive, resulting in an inflated type I error rate. Conversely, If the treatment is an active one but the trial is conducted at a location with a negative random effect, the observed data will be more likely to fail to show the difference at the desired significance level, hurting the statistical power.  As an illustration, consider the following example: suppose that we wish to test the efficacy of a treatment through some numeric response at a centre of choice out of 30 candidate locations. At any given location, the within-centre variance is 〖1.25〗^2, with an additional between-centre variance of 〖0.1〗^2 (this is akin to sampling from a cluster randomised trial with an intracultural cluster correlation (ICC) of 〖0.1〗^2 \/(〖0.1〗^2+〖1.25〗^2 )=0.006). If the 500-patient large trial is conducted across all 30 centres and analysed accordingly, the two-tailed type I error rate will be 5%. If, however, the trial is limited to the first centre – and is therefore analysed as a simple RCT – the resulting type I error rate for the global hypothesis, that is: the nation-wide efficacy – becomes as high as 22.5%. Similarly, if the true mean difference in response is 0.315, the power under the multi-centre design will be 80%, as opposed to 70% under the single centre trial. These differences are illustrated in this Figure of the main manuscript, by the distributions of the test statistics used to test for mean differences between the two treatments under both the null and the alternative hypothesis.
